# Biocontrol of *Rhizoctonia solani via* Induction of the Defense Mechanism and Antimicrobial Compounds Produced by *Bacillus subtilis* SL-44 on Pepper (*Capsicum annuum* L.)

**DOI:** 10.3389/fmicb.2019.02676

**Published:** 2019-11-28

**Authors:** Zhansheng Wu, Yuanyuan Huang, Yan Li, Jiawei Dong, Xiaochen Liu, Chun Li

**Affiliations:** ^1^Department of Bioengineering, School of Environmental and Chemical Engineering, Xi’an Polytechnic University, Xi’an, China; ^2^Department of Environmental and Biological Engineering, School of Chemistry and Chemical Engineering, Shihezi University, Shihezi, China; ^3^Department of Biochemical Engineering, School of Chemistry and Chemical Engineering, Beijing Institute of Technology, Beijing, China

**Keywords:** pepper, *Bacillus subtilis*, signaling pathway, induced systemic resistance, antimicrobial compounds, biological control

## Abstract

Pepper seedling wilt disease is the main cause of crop yield reduction. Biocontrol agents are widely used to control plant diseases caused by pathogenic fungi and activate plant defense systems. Our preliminary work showed that *Bacillus subtilis* SL-44 played a significant role in the reduction of wilt disease severity on pepper plants. To evaluate biological control mechanism of *B. subtilis* SL-44 on wilt disease caused by *Rhizoctonia solani*, the activities of the related enzymes were detected in the pepper seedling with different treatment in this study. Fluorescence microscopy combined with different dyes showed that *B. subtilis* SL-44 induced a large amount of active oxygen and callose accumulation in pepper leaves. The defense-related enzyme activities in pepper were improved significantly when treated with *B. subtilis* SL-44, including peroxidase, catalase, superoxide dismutase, polyphenol oxidase, and phenylalanine ammonia lyase. The activity of chitinase and β-1,3-glucanase in *B. subtilis* SL-44-treated pepper was also enhanced. Furthermore, the expression level of pepper-resistance gene *CaPIN II* was significantly increased in *B. subtilis* SL-44 treatment. Besides, *B. subtilis* SL-44 filtrate led to the death of the pathogenic fungus by fracturing the mycelia and leaking of the cell contents. Surfactin, iturin, and fengycin were found in *B. subtilis* SL-44 crude extracts, which could be effective antifungal compounds against *R. solani*. The results suggest that *B. subtilis* SL-44 could not only activate induced systemic resistance of pepper seedling against wilt disease caused by *R. solani* by jasmonic acid-dependent signaling pathway but also produce antifungal compounds to inhibit or even damage the mycelium growth of *R. solani*. The findings of this study provide novel guidance in plant protection development.

## Introduction

Pepper is an important cash crop with high nutritional and medicinal value (Sundaramoorthy et al., 2012). It contains various forms of vitamins (such as vitamin A, B, C, E, K), mineral substances, dietary fibers, and natural pigments that are good for human health (Mishra et al., 2017). However, the growth and production of pepper are affected by indigenous pathogens resulting in pepper yield reduction. Wilt disease caused by *Rhizoctonia solani* is responsible for pepper yield losses. Usually, these pathogens are controlled by chemical pesticides, which cause environmental pollution and potential drug resistance from long-time usage. In organic agriculture, there are restrictions on the use of more pesticides. Therefore, the application of biological control in the prevention of phytopathogen infection has gradually gained attention because it is environment-friendly. In our previous works, *Bacillus subtilis* SL-44 (SL-44) has been studied with strong antifungal activity against the mycelial growth of pathogenic fungus *Rhizoctonia solani*. Furthermore, SL-44 also has the significant capability of phosphate dissolving, IAA producing, and root colonizing and could significantly increase pepper’s biomass and promote the growth of the pepper (Huang et al., 2017). However, the discovery and understanding of SL-44 on the biological control mechanism of pepper have not been fully described on a molecular level.

It is reported that beneficial bacteria can inhibit phytopathogenic fungi by several strategies inducing cellular defense responses of plants, including cell wall thickening (Benhamou et al., 1996), active oxygen bursts (Lu et al., 2017), callose deposition, and defense-related enzyme accumulation (Yang et al., 2011). To survive in the adverse environment, plants have to evolve a variety of defense mechanisms that enable them to avoid tissue damage when pathogens attack. Systemic acquired resistance (SAR) and induced systemic resistance (ISR) are involved in plant systemic immunity. SAR is a salicylic acid (SA)-mediated broad-spectrum disease-resistance response of plants to pathogens, which usually was triggered by necrotic pathogenic bacteria (Stout et al., 1999). While, ISR was triggered by beneficial microorganisms such as plant growth-promoting rhizobacteria (PGPR) to regulate jasmonate (JA)- and ethylene (ET)-dependent signaling pathways for enhancing plant immunity rather than directly activate plant defense (Bostock, 2005; Van Wees et al., 2008; Pieterse et al., 2009). Induction of plant defenses is a new biological method for the control of plant disease. Many *Bacillus* spp. are investigated for inducing plant systemic resistance to resist pathogenic fungi. There are obvious evidences in systemic activity of defense-related enzymes such as peroxidase (POD), superoxide dismutase (SOD), polyphenol oxidase (PPO), and phenylalanine ammonia lyase (PAL) and expression of defense-related genes enhanced by *Bacillus* sp. in soybean, tomato, and *Arabidopsis thaliana* (Niu et al., 2011; Kurabachew and Wydra, 2014; Chandrasekaran and Chun, 2016; Jain et al., 2017). Differences in the expression of *Capsicum annuum* pathogenesis-related protein (CaPR) genes indicate that plant systemic immunity is elicited through SA or JA signaling pathway by different treatments (Yang et al., 2011). The main purpose of this study was to describe the effect of SL-44 on pepper plant seedling against wilt disease caused by *Rhizoctonia solani* using different strategies.

In order to reveal the mechanisms of pepper systemic resistance induced by SL-44, we characterized the defense-related enzyme activities in pepper cellular defense response. We also measured the expression levels of pathogen-associated proteins such as β-1,3-glucanase and chitinase and defense-related genes. In addition, we investigated the antifungal mechanisms of SL-44 by determining the antimicrobial compounds produced by SL-44, which is considered as a potential factor for inducing pepper’s systemic resistance or inhibiting or damaging the pathogen *R. solani*. The results of this study show that biological control of wilt disease in pepper seedling caused by *Rhizoctonia solani via* induction of the defense mechanism and production of antimicrobial compounds by *Bacillus subtilis* SL-44 gives an insight into the interaction of plant-microbe mechanism and provides a vast potential for future development in plant protection.

## Materials and Methods

### Microorganisms and Plant Materials

The pathogenic fungus *Rhizoctonia solani* was obtained from Agricultural College of Shihezi University. *R. solani* was incubated on PDA plate for 3–4 days at 28°C. The biocontrol strain *B. subtilis* SL-44 was previously originated from the rhizosphere of tomato with *R. solani* infected in Shihezi of Xinjiang in China. SL-44 was inoculated into NB (Nutrient Broth) medium for growth at 37°C, 180 rpm for 24 h in a shaker until it reached 10^8^ cfu/ml (Huang et al., 2017). Pepper seeds (*Capsicum annuum* L., *Kebao*) were purchased from a local market in Shihezi, Xinjiang of China.

Pepper seed surface was sterilized with 6% sodium hypochlorite, then washed four times with sterile distilled water, and then kept at 25°C for 3 days, until germinated on sterilized filter paper. A 7-day-old pepper seedling was transferred into a flowerpot (9 cm in diameter, 12 cm depth) containing a sterile vermiculite and perlite mixture (6:4, v:v) and placed in a growth chamber (25°C, 12/12 h photoperiod, 80–85% relative humidity). Eight plants were found in each tray and eight trays per treatment. Water and 30 ml Hoagland’s solution were added regularly for normal growth.

The 35-day-old pepper seedlings were treated in six treatments: CK (control group treated with water), SL-44 (inoculated with SL-44), R.s (inoculated with *R. solani*), SL&R.s (SL-44 inoculation and *R. solani* infection), SA (pepper foliage treated with 0.5 mM salicylic acid), and MeJA (pepper foliage treated with 0.1 mM methyl jasmonate). Method of microorganism inoculation is as follows: 5 ml per flowerpot of the overnight cultured SL-44 broth was pipetted into the 2 cm depth soil around root of the pepper seedlings with a sterile syringe; the mature *R. solani* hyphae cultured in the potato dextrose liquid medium for 5 days was taken out, and the hyphae ball with a diameter of about 1 cm was selected and buried at a depth of 2 cm around the root of the pepper seedling; when pepper seedling was inoculated with both biocontrol bacteria and plant pathogens, the fungus was inoculated at first; and staged spray 5 ml of salicylic acid at a concentration of 0.5 mM onto the leaves of the pepper on each flowerpot. After salicylic acid was completely absorbed by leaves, it can be used as samples of SA treatment. The method of MeJA treatment was same with SA treatment but concentration of methyl jasmonate was 0.1 mM.

### Detection of Hydrogen Peroxide and Callose by Histochemistry and Fluorescence Staining

Histochemical and fluorometric staining assays were conducted in different treatment of pepper plant 24 h post inoculation (hpi). Six mature leaves are collected and tested by 3, 3′-diaminobenzidine (DAB) staining to detect H_2_O_2_ and aniline blue staining to detect callose (method by Thordal-Christensen et al., 1997). Three repeats of every treatment were carried out.

The leaves of pretreated were stored in a 50% (v/v) ethanol solution for microscopic examination. The coloration was observed under a 40 × magnification using a ZEISS optical microscope and photographed.

According to the method of Conrath et al. (1998), the pretreated leaves were stored in distilled water for microscopic examination. The OLYMPUS BX51TR fluorescence microscope was used to observe the coloration under ultraviolet excitation light (*λ* = 385 nm) and photographed.

### Defense-Related Enzyme Activity Detection

Samples were collected 1, 3, 5, 7 and 9 days after inoculation (dai). About 0.5 g of pepper leaves of fresh weight was ground in a pre-cooled mortar. The grounded powder was added into 3 ml different PBS buffers with the corresponding pH required for different enzyme activity assays. The homogenate was centrifuged at 10,000 × *g* for 20 min at 4°C. The crude enzyme extracts (supernatants) were collected and stored in a refrigerator at −20°C for detection.

The method of activity detection of SOD was photoreduction of nitroblue tetrazolium (NBT). One SOD enzyme activity unit refers to the amount of enzyme needed to inhibit 50% NBT photoreduction reaction. The method of guaiacol was used for POD detection, 0.01 increase in A_470_ per minute is a POD enzyme activity unit. The activities of catalase (CAT), PAL, and PPO were tested by UV absorption, the amount of enzyme that reduces the A_240_ by 0.1 per minute is CAT enzyme activity unit. The amount of enzyme that changes A_290_ by 0.01 per minute is PAL enzyme activity unit. The increase of A_398_ by 0.01 per minute was defined as a PPO enzyme activity unit. All of these enzyme activity detection methods were according to Gao (2006). In each of the enzyme studies, each treatment is composed of six replicates and spectrophotometric readings using a UV-VIS Spectrophotometer (UV 752 N, YUANXI, Shanghai).

### Detection of β-1,3-Glucanase and Chitinase in Pepper Leaves

About 0.5 g of treated pepper leaves (3 dai) was placed in a pre-cooled mortar and quickly ground to powder with liquid nitrogen protection; 5 ml of 0.05 M acetic acid-sodium acetate buffer (pH 5.0) was added to the extract and then centrifuged at 8,000 × *g* for 30 min at 4°C; and the crude enzyme extract obtained was stored in the refrigerator at −20°C until use.

Boller and Mauch’s (1988) method was used for chitinase activity detection. OD_540_ was determined by UV-VIS Spectrophotometer (UV 752 N, YUANXI, Shanghai) and repeated three times for each treatment.

Pan et al.’s (1991) method was used for β-1,3-glucanase detection. The absorbance of the mixture was determined at 540 nm by UV-VIS Spectrophotometer (UV 752 N, YUANXI, Shanghai). The experiment was repeated three times.

### Determination of Transcripts Levels of Induced Systemic Resistance Defense-Related Gene

RNA samples were isolated from four treated pepper leaves using real-time polymerase chain reaction (RT-PCR) to verify the differential expression of selected genes. Expression of *C. annum pathogenesis-related 1* (*CaPR1*), *C. annum basic β-1,3-glucanase* (*CaPR2*), *C. annuum pathogenesis-related protein 4* (*CaPR4*), and *C. annuum proteinase inhibitor II* (*CaPIN II*) (Table 1) have been previously reported to be related to the defensive responses (Marrs, 1996; Shin et al., 2001; Park et al., 2002; Yang et al., 2011). CaACT1 was used as an internal reference gene to calibrate the expression of other mRNA genes (GenBank accession no. AY572427).

**Table 1:** Primers used in this study.

Gene	Primer name	Primers sequences	Sources
*CaACT1*	ACT1-F ACT1-R	CAATCCCTCCACCTCTTCAC ATCCAGCCTTAACCATTCCTG	Yang et al. (2011)
*CaPR1*	PR1-F PR1-R	AGCCCAAAATTCTCCCCAAG CTAGCCTATTGTCCCATGTCATAG	Yang et al. (2011)
*CaPR2*	PR2-F PR2-R	TGTGAAATGAAGTCAGCCCTG TCCGAATGTTTCTCATGGCAG	Marrs (1996)
*CaPR4*	PR4-F PR4-R	GGTAGATGCTTGAGGGTGAC CCGTCGATCAGTGTCCAATTG	Park et al. (2002)
*CaPIN II*	PIN II-F PIN II-R	CCGAAGGAAACGCAGAAAATC GTCCCGATGACGCTGTAATAG	Shin et al. (2001)

Total RNA was isolated using RNAprep Pure Plant Kit (TIANGEN, China). The first strand cDNA was reverse transcribed using PrimeScript™ RT reagent Kit with gDNA Eraser (Takara Bio. Inc., Japan). The Oligo(dT) primer was synthesized by GENEWIZ (Suzhou, China).

Quantitative RT-PCR reactions were performed on the Light Cycler 96 system (Roche, USA) using SYBR®*Premix Ex Taq*™ II according to the instruction. The extracted sample to be tested in condition of pre-denatured at 95°C for 30 s, and then the PCR cycle is started, and followed by 40 cycles. Dissociation curve analysis at 95°C for 0 s, 65°C for 15 s, and 95°C for 0 s. All genes evaluated were identical for the qRT-PCR parameters. The qRT-PCR experiments were conducted twice with three replicates for each treatment.

### Effect of Culture Filtrates of *Bacillus subtilis* SL-44 on *Rhizoctonia solani* Mycelial Growth

To detect the disruption of the culture filtrate of SL-44 against the pathogenic fungus *R. solani*, SL-44 was inoculated into 50 ml NB medium and placed in a shaking incubator with 170 rpm at 30°C. After culturing for 36 h, the bacterial suspension was collected and centrifuged at 10,000 × *g* for 10 min at 4°C. The supernatant was filtered using a 0.22 μm filter. *R. solani* was inoculated on PDA, grown, and covered with agar medium surface, and then we used a 10-mm diameter punch holes in the fungus-coated medium to obtain a 10-mm diameter mycelial disk of *R. solani*. About 10-mm diameter mycelium of *R. solani* was mixed with SL-44 filtrate and incubated at 28°C for 24 h. The same size of mycelium was added into sterile water as a control. The experiment was repeated three times for each treatment. The sample was observed under an optical microscope (ZEISS, Germany) to observe changes in the mycelium.

### Isolation and Identification of Antimicrobial Compounds From *Bacillus subtilis* SL-44 Filtrates

Five milliliters of activated SL-44 culture was inoculated into 200 ml LB medium and cultured at 30°C for 36 h. The cultured SL-44 broth was first centrifuged (4°C, 12,000 × *g* for 20 min) and removed the cells, the supernatant was filtered using a 0.22 μm sterile filter, and then the filtered supernatant was mixed with the equal volume of n-butanol. The antibacterial lipopeptide in the supernatant was extracted into the n-butanol phase for three times, then concentrated with rotary evaporation to dry at 50°C, and residual was dissolved in a small amount of sterile water prior to use (Huang et al., 2014).

Iturin A in the antimicrobial crude extract was identified by reversed-phase high-performance liquid chromatography (HPLC, LC-A10, Shimadzu, Japan) with an ultraviolet detector. A reversed phase column Agilent ZORBAX Eclipse Plus C18 (4.6 mm × 250 mm, 5 μm) was used and the detection wavelength was 230 nm. Mobile phase A was HPLC grade methanol and B was 0.1% acetic acid solution, A:B = 40:60 at a flow rate of 0.8 ml/min at 40°C.

To further determine lipopeptides in antimicrobial crude extracts, the molecular weight of the crude extracts was determined by LC-MS/MS (HPLC: Agilent 1,290, USA, MS/MS: TSQ Quantum Ultra, USA). The column was a liquid column Thermo HYPERSIL GOLD C18 (100 mm × 2.1 mm, 1.9 μm). Mobile phase A was a 0.1% formic acid solution and mobile phase B was 100% acetonitrile at a flow rate of 0.3 ml/min. Mass spectrometry detection conditions are as follows: cone voltage 3 kV, electrospray temperature 300°C, capillary temperature 350°C, and scan range 800–1,600 m/z. Data were acquired using a positive ion mode.

### Statistical Analysis

All data were presented using mean ± standard deviation (SD). Significant differences between the means were determined using one-way analysis of variance (ANOVA) and Duncan’s multiple range tests (*p* < 0.05) to compare the difference in enzyme activity of control and treatments and defense-related gene expression of different treatments in the same time intervals (*p* < 0.05, SPSS 18.0 statistical software. IBM Corp., Armonk, NY).

## Results

### *Bacillus subtilis* SL-44 Stimulated Hydrogen Peroxide Accumulation and Callose Deposition

**Figure 1:**
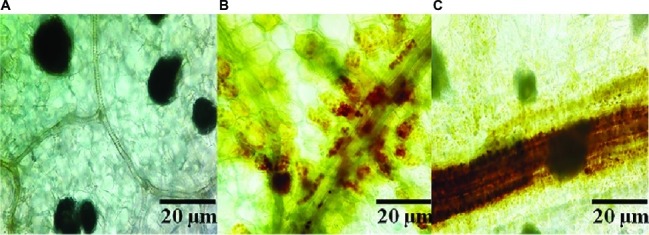
Production of ROS in pepper foliage in different treatments of control **(A)**, *Rhizoctonia solani* **(B)**, and SL-44 **(C)**.

In order to clarify the mechanism of potential activation of cell defense responses in pepper plants by SL-44, the accumulation of H_2_O_2_ in different treatments of pepper foliage was examined. Compared to the control treatment (Figure 1A), reddish-brown precipitates were obviously observed in both *R. solani* (Figure 1B) and SL-44 (Figure 1C) treatments, respectively. In addition, the pepper leaves which were treated with SL-44 showed a darker color of reddish-brown precipitates than those treated with pathogenic fungus at the same time. H_2_O_2_ production was detected in pepper foliage in two treatments, indicating that the addition of exogenous microorganisms primed a cellular defense reaction produced in pepper plant.

**Figure 2:**
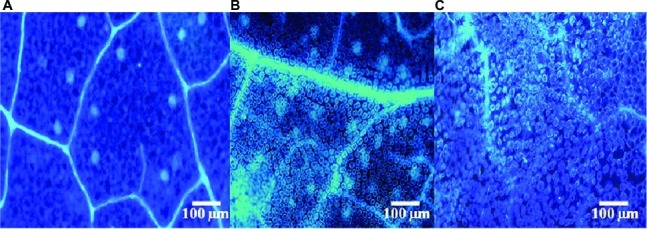
Fluorescent staining of callose of pepper foliage in different treatments of control **(A)**, *Rhizoctonia solani* **(B)**, and SL-44 **(C)**.

To assess whether SL-44 activates the pepper defense responses, callose deposition in peppers was assessed (Figure 2). A clear blue fluorescence was observed both in *R. solani* (Figure 2B) and SL-44 (Figure 2C) treatments when compared with water treatment with almost no fluorescence observed (Figure 2A). Treatments with *R. solani* (Figure 2B) and SL-44 (Figure 2C) led to an increase in callose deposition in leaves at 24 hpi, indicating that the addition of exogenous microorganisms could cause the accumulation of callose in the leaves of pepper plants for producing a defense reaction of plant cells.

### Induction of the Activity of Superoxide Dismutase, Peroxidase, Catalase, Phenylalanine Ammonia Lyase, and Polyphenol Oxidase in Pepper Leaves by *Bacillus subtilis* SL-44

**Figure 3:**
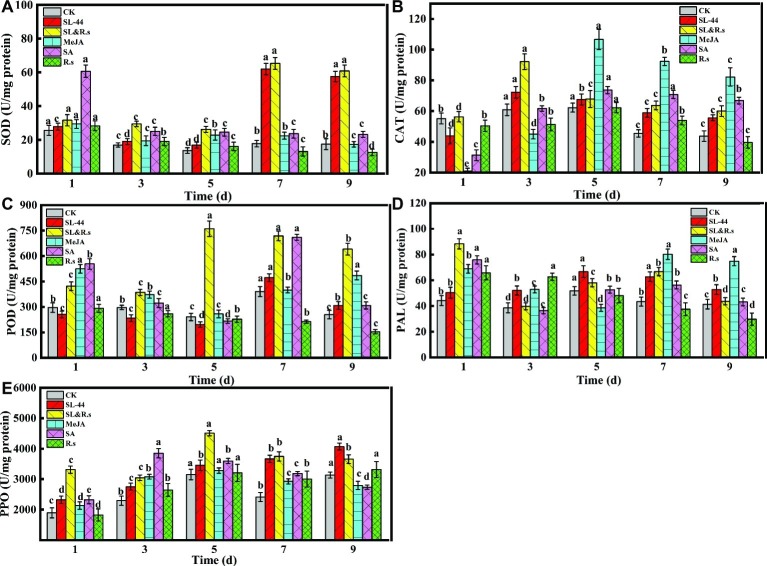
Activities of different defense-related enzymes of pepper foliage. **(A)** SOD, **(B)** POD, **(C)** CAT, **(D)** PAL, and **(E)** PPO. Each value represents the mean ± SD (*n* = 6). Different letters above the columns indicate significant differences at *p* < 0.05.

The defense enzyme activities of SOD, POD, CAT, PAL, and PPO were determined to investigate the effect of induced resistance on pepper by SL-44. The activities of SOD, POD, CAT, PAL, and PPO in pepper leaves reached maximum at 7, 7, 3, 5, and 9 dai in SL-44 treatment, which were 71.3, 17.3, 15.8, 22.3, and 23% better than that of CK treatment (Figure 3), respectively. Meanwhile, SOD (Figure 3A), CAT (Figure 3B), PAL (Figure 3D), PPO (Figure 3E), and POD (Figure 3B) activities of SL-44 treatment were 63.7, 15.4, 37.6, 42.1, and 31.4% higher than that of MeJA treatment, and also SOD, CAT, PAL, and PPO of SL-44 treatment were 61.8, 14.7, 21, and 32.9% better than that of SA treatment, respectively. While POD activity in SL-44 treatment was 33.5% lower than that of in SA treatment. In addition, the enzyme’s activity of SL&R.s treatment was also significantly higher than that of CK, which were 72.8, 68.3, 33.9, 49.8, and 31% higher than CK in 7, 5, 3, 1, and 5 dai, respectively. The pepper defensive enzymes activity decreased significantly with increasing inoculation time of R.s. such as SOD, POD, and PAL, while some enzyme activities such as CAT and PPO showed a first increase and then decrease in different inoculation time.

In this study, all enzyme activities were significantly increased in SL-44 treatment compared to CK treatment at 7, 7, 3, 5, and 9 dai, respectively (Figure 3). MeJA and SA treatments as a positive control to active ISR and SAR in pepper, respectively. When all enzyme activity reached the maximum in SL-44 treatments at 7, 7, 3, 5, and 9, these enzyme activities of pepper make SL-44 treatment even higher than that in MeJA and SA treatments. The above results indicate that SL-44 induces the enhancement of SOD, POD, CAT, PAL, and PPO activities thereby protecting pepper plant against pathogen infection.

### Chitinase and β-1,3-Glucanase in Pepper Leaves

**Figure 4:**
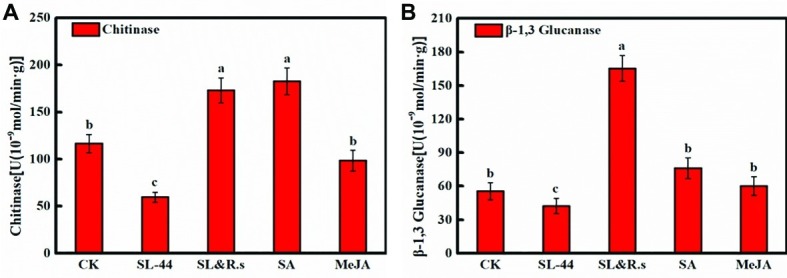
Chitinase and β-1,3-glucanase of pepper foliage in different treatments of chitinase activity **(A)** and β-1,3-glucanase activity **(B)**. Each value represents the mean ± SD (*n* = 6). Different letters above the columns indicate significant differences at *p* < 0.05.

The activities of chitinase and β-1,3-glucanase reflect the ability of plants to resist external aggressions. In this study, the effect of plant pepper resistance inoculated with SL-44 was evaluated by testing chitinase and β-1,3-glucanase activities. As shown in Figures 4A,B, chitinase activity of SL-44 treatment was significantly lower than that of CK, SL&R.s, SA, and MeJA treatments by 48.9, 65.6, 67.4 and 39.6%, respectively. While, β-1,3-glucanase activity was significantly lower than that of CK, SL&R.s, SA, and MeJA treatment by 23.7, 74.3, 44.3 and 29.5%, respectively. Chitinase and β-1,3-glucanase activities in pepper foliage were not enhanced by SL-44 treatment (Figure 4). This result showed that SL-44 could not induce plant pepper systemic acquired resistance (SAR) by activating SA-dependent signaling pathways.

### Relative Expression of Systemic Acquired Resistance and Induced Systemic Resistance Defense-Related Genes

**Figure 5:**
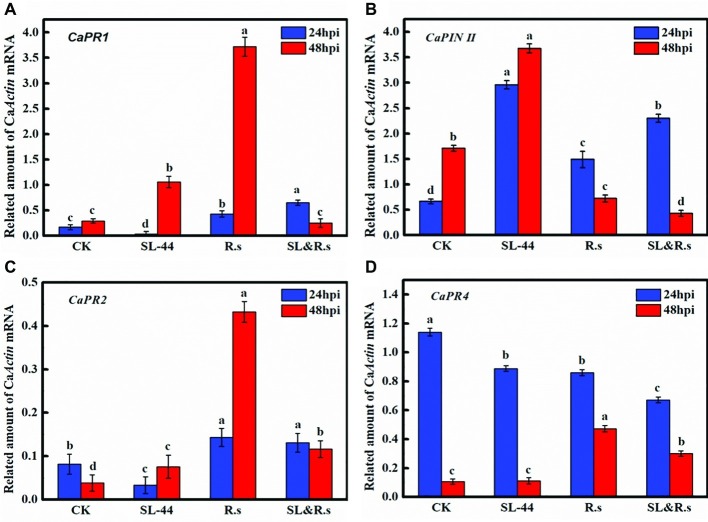
Expression level of the different defense-related gene of pepper foliage in different treatments. **(A)** *CaPR1*, **(B)** *CaPIN II*, **(C)** *CaPR2*, and **(D)** *CaPR4*. Each value represents the mean ± SD (*n* = 3). Different letters above the columns indicate significant differences at *p* < 0.05.

Induction of pepper defense genes by SL-44 was assessed in foliage using RT-PCR. The transcription of basic β-1,3-glucanase (*CaPR2*) in pepper leaves treated with SL-44 was lower than those treated with water in 24 hpi (Figure 5C). There was no significant increase in *CaPR1* and *CaPR4* transcription of pepper in SL-44 treatment when compared with other treatments (Figures 5A,D). While the expression level of *CaPIN II* in pepper treated with SL-44 was significantly higher than that of other treatments (Figure 5B).

### *Rhizoctonia solani* Suppression Using *Bacillus subtilis* SL-44 Culture Filtrates

**Figure 6:**
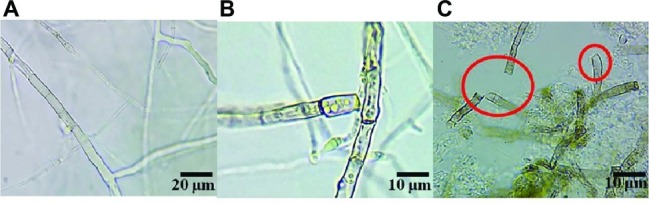
The strain SL-44 suppressed mycelial growth of *Rhizoctonia solani* in different treatments of control **(A)**, 16 hpi **(B)**, and 24 hpi **(C)** treated with SL-44 culture filtrates.

The destruction of the mycelium growth of *R. solani* by the SL-44 filtrate was examined by light microscopy. The results showed high vascularization, protoplasm leakage, cell wall damage and breakage in pathogens when compared to the normal growth of the control (Figure 6A) when the pathogenic hyphae were treated with SL-44 filtrate for 16 h (Figure 6B). When treated for 24 h, a large number of irregular growth, distortion, and fracture of the pathogenic mycelium of *R. solani* were obviously observed (Figure 6C).

### Preliminary Detection and Species Analyses of Antimicrobial Compounds Produced by *Bacillus subtilis* SL-44

**Figure 7:**
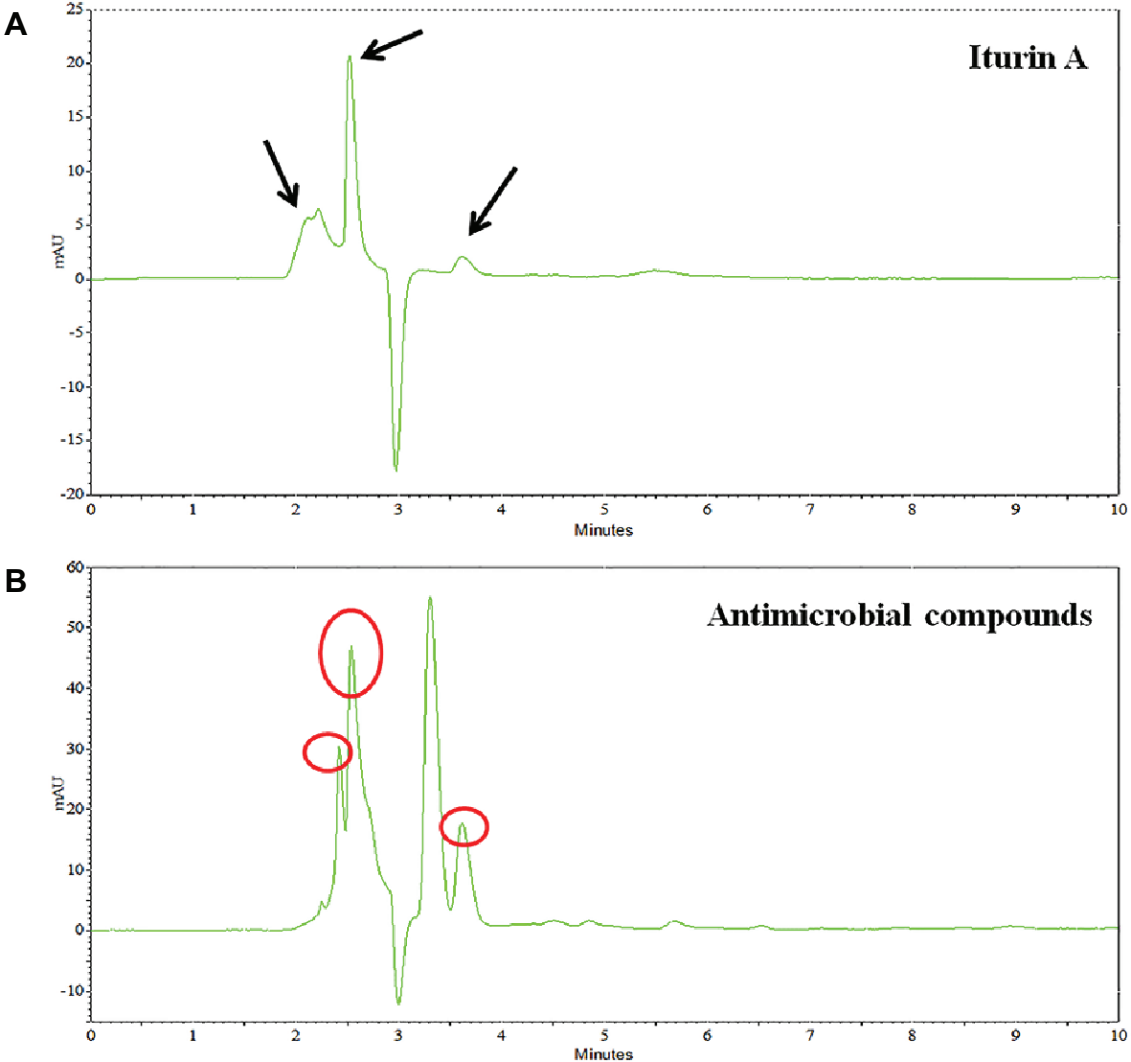
HPLC preliminary identification of iturin A **(A)** and crude extracts of SL-44 **(B)**.

Preliminary identification of the SL-44 antibacterial crude extract was performed by HPLC compared with the iturin A standard. The results are shown in Figure 7. The iturin A standard consists of three homologues with similar structures but different molecular weights. The presence of iturin A in crude extract samples was identified by comparison of the peak times with standard chromatograms (Figure 7A). The signal peaks with the same retention time in the two liquid chromatograms were preliminarily considered to be the same substance. There are also three suspected iturin A substances present in the n-butanol extract at 2.251, 2.539, and 3.616 min (Figure 7B), respectively. They all exhibited absorption peaks at 230 nm. Therefore, the initial identification of SL-44 antibiotic substance could contain iturin A.

**Figure 8:**
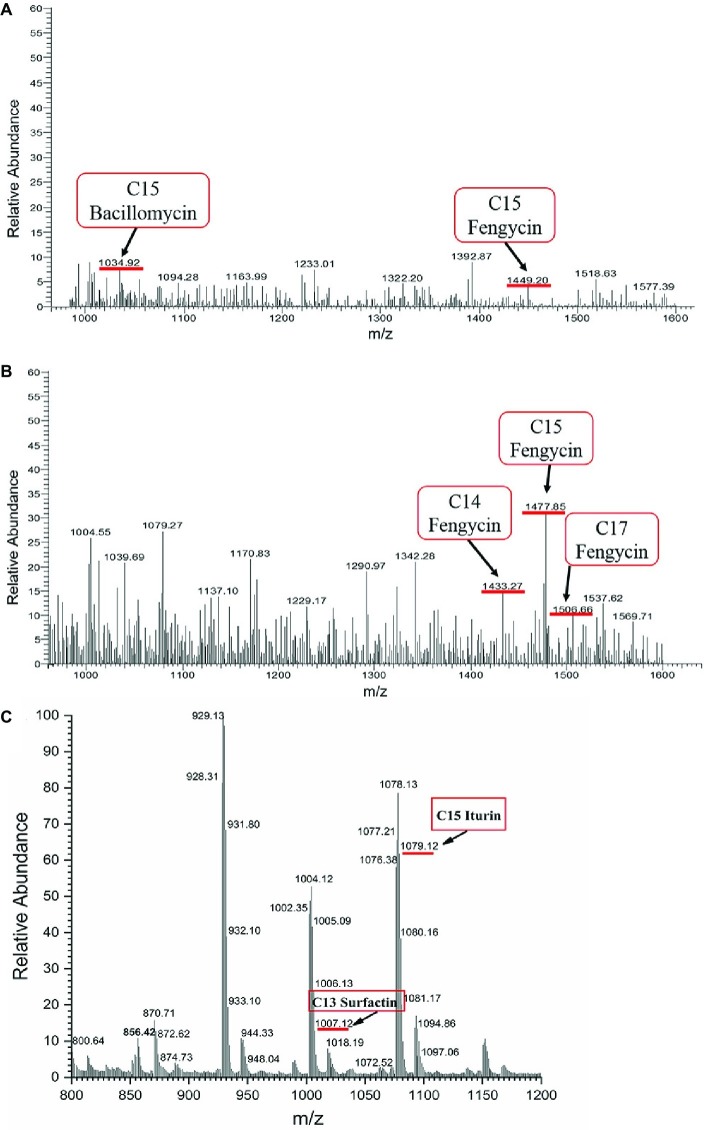
LC-MS/MS analyses on SL-44 crude extracts in different signal peak retention time: **(A)** 3.72 min, **(B)** 7.35 min, and **(C)** 11.89 min.

The crude extract of SL-44 was also analyzed by LC-MS/MS, and the results of the molecular weight determination are 1007.12, 1034.92, 1079.12, 1433.27, 1449.20, 1477.85, and 1506.66. Each set of peaks can be attributed to different lipopeptide isoforms compared with the mass number previously reported for lipopeptide complexes from other *Bacillus* strains. Each isoform may have the same amino acid sequence, but the length of the fatty acid chain is different. Among them, 1007.12 is consistent with the molecular weight of surfactin A (C13) (Figure 8C); 1034.92 and 1079.12 correspond to the molecular weights of bacillomycin L (C15) and iturin A (C15) (Figures 8A,C), respectively, while 1433.27, 1449.20, 1477.85, and 1506.66 correspond, respectively, to fengycin A (C14, 6-Ala) (Figure 8B), fengycin A (C15, 6-Ala) (Figure 8A), fengycin B (C15, 6-Val) (Figure 8B), and fengycin B (C17, 6-Val) (Figure 8B) and are consistent (Wang et al., 2004; Luo et al., 2015; Mnif et al., 2015). Based on the above results, the antibacterial crude extract of SL-44 contain surfactin A, iturin substances (including bacillomycin L and iturin A), and fengycin A and fengycin B substances.

## Discussion

The root rot caused by *R. solani* in the world has become an urgent problem to be solved. The use of chemical fungicide to control this disease has been limited because of drug resistance, environmental pollution, and restricted for use in organic agriculture. In previous study, the use of SL-44 as a biological control agent and its effect on *R. solani* were examined and the result obtained was promising (Huang et al., 2017). This work is expected to further study the biocontrol mechanisms of SL-44 on pepper and against *R. solani*. SL-44 was found to induce resistance in pepper and produce lipopeptides against *R. solani*. And this is the first time to extensively illustrate the biocontrol mechanisms of SL-44 on pepper against *R. solani*.

*B*. *altitudinis* JSCX-1 can effectively reduce the occurrence of *Phytophthora sojae* by increasing the accumulation of reactive oxygen and callose in soybean (Lu et al., 2017). Wild-type *Arabidopsis thaliana* Col-0 was inoculated with *B. cereus* AR156, which also activated plant defense responses and detected the accumulation of large amounts of H_2_O_2_ and callose in plant leaves (Niu et al., 2011). Similarly, Wu et al. (2018) reported that H_2_O_2_ and callose in *Arabidopsis thaliana* play a key role in the early stage of defense responses against *Verticillium dahliae*, which is consistent with the results of this study. In Figures 1, 2, SL-44 caused massive accumulation of H_2_O_2_ and callose deposition, which showed that SL-44 activates pepper defense reactions to pathogens.

In the interaction of plant-pathogens, ROS plays a dual role. ROS can stimulate host disease resistance response, yet the accumulation of active oxygen in excess of a certain amount will also damage the host’s own cells. Fortunately, a series of antioxidant enzymes are used to scavenge ROS in plant, such as SOD, POD, and CAT (Chandrasekaran and Chun, 2016). PAL can synthesize phenols, lignin, and other substances that are associated with disease resistance by catalyzing the key enzyme of phenylalanine and play an important role when plants are attacked by pathogens. PPO can participate in the oxidation of phenolic compounds and synthesize anthraquinones that passivate pathogens (Li et al., 2015). As an endogenous signal molecule, MeJA can participate in plant response to pathogen and other adverse stresses and signal transmission, and it also can be used as an exciton to induce plant disease resistance (Zhang et al., 2010). SA is an important pathogenic signal molecule, and it can also induce the expression of pathogenesis-related proteins (PRs) genes and enhances plant resistance to pathogen (Mandal et al., 2009). Similar studies have been reported, when two strains of *B. subtilis* EPCO16 and EPC5 were mixed and then inoculated into the pepper. Pepper was found to be a good resistance to (Sundaramoorthy et al., 2012). It was reported that the different defense-related enzymes such as PPO, POD, and PAL were the highest in *Glycine max* L. Merrill and were clearly expressed in the root tissue on the 8th day treated with *Bacillus* SJ-5 after *R. solani* and *Fusarium oxysporum* challenge inoculation (Jain et al., 2017). *Bacillus subtilis* CBR05 may play a key role in alleviating ISR oxidative stress by increasing the activity of enzymes and antioxidant enzymes (SOD, POD, CAT, PPO and PAL) in early defense against bacterial soft rot in tomato (Chandrasekaran and Chun, 2016). Similar results were obtained in pepper plant of SL-44 treatment, suggesting SL-44 increased the defense-related enzyme activity and activated cellular defense response thereby inducing pepper systemic resistance (ISR) against pathogens.

Beneficial microbes can induce plant systemic resistance (ISR) by activating two signaling pathways, the JA/ET-dependent (major) signaling pathway and the SA-dependent (minor) signaling pathway. When the SA-dependent signaling pathway is activated, the contents of pathogenesis-related (PR) proteins, such as chitinase and β-1,3-glucanase, will increase in the plant. It was reported that *B. subtilis* can increase expression of PR protein such as β-1,3-glucanase as well as induce tomato systemic resistance against soft rot disease (Chandrasekaran and Chun, 2016). In the present study, SL-44 may activate the pepper systemic resistance by JA/ET-dependent (major) signaling pathway. Further experiments should be performed to verify the special findings in the current study.

Pathogenesis-related genes *CaPR1*, *CaPIN II*, *CaPR2,* and *CaPR4* were discovered from pepper (*Capsicum annuum* L.) by researchers (Marrs, 1996; Shin et al., 2001; Park et al., 2002; Yang et al., 2011). *CaPR1* is closely related to the SA-responsive signaling pathway, *CaPIN II* is mainly for JA-responsive signaling, *CaPR2* is for ET/SA signaling, and *CaPR4* is for JA/ET signaling. The transcription level of *CaPR1*, *CaPR2,* and *CaPR4* in pepper foliage treated with SL-44 had no significant difference compared to that of CK treatment (Figures 5A,C,D). It is worth mentioning that *CaPR2* is a gene that controls β-1,3-glucanase expression, while it has not been significantly expressed in the SL-44 treatment (Figure 5C). However, the expression level of *CaPIN II* of SL-44 treatment was noticeably higher than that of CK, R.s, and SL&R.s treatments (Figure 5B). The results above showed that SL-44 cannot induce increase activity of β-1,3-glucanase in pepper (Figure 4B). However, SL-44 stimulates JA-dependent signaling pathway rather than ET-dependent signaling pathway in ISR mechanisms. There are several studies, which prove that microorganisms could stimulate JA-dependent signaling pathway in ISR mechanisms. In the Whitefly-induced resistance gene expression analysis of pepper, the gene expression of *CaPR1*, *CaPR4*, *CaPR10,* and *CaPIN II* was significantly increased after the treatment with benzothiazole (BTH) and/or whitefly, which indicated that SA and JA signaling pathways in AG and below ground (BG) were induced by above-ground (AG) whitefly infestation (Yang et al., 2011). It has been reported that the same genes (*CaPR1*, *CaPR4*, *CaPR10*, and *CaPIN II*) were not only activated both in SAR and ISR systems that are incompatible in pepper but also showed significant difference on expression between the whitefly-treated and control group except *CaPR4* gene (Yang et al., 2009). The transcription of *CaPR2* was increased when pepper was soaked in 2,3-butanediol produced by *B. subtilis*, which suggested that the strain activated plant defenses mainly *via* SA and ET signaling pathways (Yi et al., 2016). The transcription of all pathogenesis-related genes under the treatment of SL-44 suggested that ISR is primed in pepper mainly *via* JA-dependent signaling pathway and pepper produced defense resistance against pathogen.

Antifungal compounds act directly on pathogens. For example, *Bacillus pumilus* can produce antifungal metabolites, which inhibit the growth of many species of mycelial such as *Penicillium*, *Aspergillus,* and *Fusarium* (Lu et al., 2017). In this work, the filtrate of SL-44 had a destructive effect on *R. solani*. It can destroy the cell wall of *R. solani* and also cause pathogenic mycelium to appear highly vacuolated, protoplasm leakage, irregular growth, distortion, and broken (Figure 6). Similarly, it has been reported that the supernatant of *B. subtilis* V26 can cause an increase of cytoplasmic vacuoles, cell wall disintegration, and protoplasm leaks in the mycelium of *R. solani*. The reason was that antifungal compounds in culture supernatant produced by *B. subtilis* V26 inhibit mycelial growth of *R. solani* or kill pathogenic fungus (Chen et al., 2016). *B. altitudinis* JSCX-1 can induce morphological changes in *Phytophthora sojae*, and the production of antifungal compounds is one of the mechanisms for controlling *Phytophthora* rot (Lu et al., 2017). *B. amyloliquefaciens* PG12 suppresses the growth of *Botryosphaeria dothidea* by damaging the mycelial growth, and the lipopeptides were considered as the main role of PG12 biological control ability (Ben et al., 2015). Morphological changes of *R. solani* mycelium caused by SL-44 were consistent with the conclusions of Ben et al. (2015) and Chen et al. (2016), which also revealed that there is a great possibility that antifungal compounds present in SL-44 filtrates play a key role in biological control mechanisms.

Several *Bacillus* spp. have been reported as biological control agents for plant diseases (Mnif et al., 2015; Ali et al., 2016; Gu et al., 2017). They have convincing antagonistic properties because these microorganisms synthesize broad-spectrum antibacterial compounds (Huang et al., 2014). Antimicrobial compounds from *Bacillus* protect plants either by directly inhibiting the pathogens or by stimulating ISR in the host (Asaka and Shoda, 1996). In this study, *B. subtilis* SL-44 showed antagonism against *R. solani* (Figure 6). This result showed that SL-44 can secrete a large number of antibacterial compounds. We attempted to isolate the antifungal compounds produced by SL-44 to explain the biological control mechanisms. By extraction, precipitation, and identification, three types of lipopeptide were observed and identified, surfactin (C13, surfactin A) (Figure 8C), iturin (C15, bacillomycin L; C15, iturin A) (Figures 8A,C), and fengycin A (C14, 6-Ala; C15, 6-Ala) (Figures 8A,B) and B (C15, 6-Val; C17, 6-Val) (Figure 8B) according to previous studies (Wang et al., 2004; Mnif et al., 2015). Liu et al. (2014) reported that three isoforms, surfactin, iturin, and fengycin can affect spore germination and membrane permeability of four fungal plant pathogens (*Alternaria solani*, *Fusarium sambucinum*, *Rhizopus stolonifer*, and *Verticillium dahliae*). Mnif et al. (2015) extracted lipopeptides, iturin subtype structural material, and fengycin from *B. subtilis* SPB1 and found that it had a strong inhibitory effect on the growth of *R. solani* and *R. bataticola*. Zhu et al. (2012) detected the antagonistic effect of lipopeptides (one of which was a surfactin) produced by *B. amyloliquefaciens* XZ-173 on *R. solanacearum* QL-Rs1115. Gu et al. (2017) reported that bacillomycin D produced by *B. amyloliquefaciens* FZB42 has an antagonistic effect on corn and wheat on *F. graminearum*. Antifungal compounds can also resist pathogenic bacteria by inducing plant resistance to prevent pathogen infection (Choudhary and Johri, 2009; Lu et al., 2017). *B. subtilis* UMAF6639 secretes lipopeptides to protect plants against powdery mildew by activating JA- and SA-dependent defense responses (García-Gutiérrez et al., 2013). In this study, the antimicrobial lipopeptides, iturin A, bacillomycin L, surfactin, and fengycin, produced by SL-44 were identified and were considered to play essential role in biological control against *R. solani* and induce pepper defense resistance such as to trigger cellular defense response, induce defense-related enzyme enhancement, and stimulate the JA-dependent signaling pathway.

In conclusion, this study gives a preliminary explanation to prevention mechanism of SL-44 against wilt disease caused by *Rhizoctonia solani* in pepper. SL-44 is a beneficial bacterium that has successfully performed the outstanding act of biological control in pepper seedling *via* two main strategies. On the one hand, SL-44 induced pepper plant systemic resistance by activating JA-mediated signaling pathway and enhanced plant immunity to defense pathogen aggression in pepper. On the other hand, it also produced lipopeptides such as fengycin and iturin that inhibit mycelium growth of *R. solani* and can also damage this pathogenic fungus. This work will provide novel guidance in plant protection development.

## Data Availability Statement

All datasets generated for this study are included in the article/supplementary material.

## Author Contributions

ZW was conceived and supervised the study as corresponding author. YH designed and performed the experiments in a major part and wrote the manuscript. YL and JD assisted for performing experiments and analyzing data. XL revised the important academic content of the article. CL assisted on supervising the study. All authors approved the final article.

### Conflict of Interest

The authors declare that the research was conducted in the absence of any commercial or financial relationships that could be construed as a potential conflict of interest.
